# 3D printers in hospitals: Reducing bacterial contamination on 3D-printed material

**DOI:** 10.1017/ash.2023.397

**Published:** 2023-09-29

**Authors:** Katelin Jackson, Douglas Call, Eric Lofgren

## Abstract

**Background:** COVID-19 has presented hospitals with unique challenges. An SHEA Research Network survey showed that 40% reported “limited” or worse levels of personal protective equipment (PPE) and that 13% were self-producing PPE to address those deficits, including 3D-printed items. However, we do not know how efficiently, if at all, 3D-printed materials can be disinfected. Additionally, 2 filaments, PLACTIVE and PUREMENT, claim to be antimicrobial; they use copper nanocomposites and silver ions to reduce bacterial populations. We assessed how PLACTIVE and PUREMENT may be contaminated and how well they reduce contamination, and how readily polylactic acid (PLA), a standard 3D-printed material, may be disinfected. **Methods:** We grew methicillin-resistant and -susceptible *Staphylococcus aureus*, *Escherichia coli*, and *Klebsiella pneumoniae* on 3D-printed disks and conducted bacterial survival assays to determine whether bacteria grow on PLA, PLACTIVE, and PUREMENT. We performed a time series (with 3- and 24-hour dry times) followed by serial dilutions to attain colony-forming unit (CFU) averages for each strain per disk. To determine whether 3D-printed material can be cleaned, we used 70% EtOH on PLA only. We conducted the same time series followed by a disinfectant time series (with dry times 30 seconds, 2.5, minutes, 5 minutes, and 10 minutes). Again, serial dilutions were performed to attain the PLA CFU averages with disinfectant. The CFU averages from the control group (PLA) and testing group (PLACTIVE and PUREMENT) were compared to see how well the antimicrobial material decreased bacterial load. We also compared the CFU averages of PLA with and without disinfectant to see how well 70% EtOH decreased bacterial load. **Results:** 3D-printed material is readily contaminated with bacteria common in hospitals and can sustain that contamination. Antimicrobial materials, PLACTIVE and PUREMENT, had lower levels of bacterial contamination when compared to PLA. However, disinfected disks had lower overall CFU averages than those that were not, but the level of disinfection was variable and bacterial populations recovered hours after disinfection application. **Conclusions:** Proper disinfection and using appropriate 3D-printed materials are essential to limiting bacterial contamination. 3D printers and their products can be invaluable for hospitals, especially when supplies are low and healthcare worker safety is paramount. Environmental services should be made aware of the presence of antimicrobial 3D-printed materials, and patients should be discouraged from printing their own items for use in hospital environments.

**Disclosures:** None

